# Sepsis Induces a Long-Lasting State of Trained Immunity in Bone Marrow Monocytes

**DOI:** 10.3389/fimmu.2018.02685

**Published:** 2018-11-19

**Authors:** Katharina Bomans, Judith Schenz, Isabella Sztwiertnia, Dominik Schaack, Markus Alexander Weigand, Florian Uhle

**Affiliations:** Department of Anesthesiology, Heidelberg University Hospital, Heidelberg, Germany

**Keywords:** SIRS, CARS, immunosuppression, metabolism, immune memory

## Abstract

Innate immune memory describes the functional reprogramming of innate immune cells after pathogen contact, leading to either a boosted (trained immunity) or a diminished (immune tolerance) response to a secondary stimulus. Immune tolerance or “sepsis-induced immunosuppression” is a typical hallmark of patients after sepsis survival, characterized by hypo-responsiveness of the host's immune system. This condition renders the host vulnerable for a persisting infection or the occurrence of secondary, often opportunistic infections, along with an increased mortality rate. The mechanisms involved in the maintenance of this long-lasting condition are not examined yet. Polymicrobial abdominal sepsis was induced in 12 week old male C57BL/6 mice by cecal ligation and puncture. Mice were euthanized 3 months after insult. Immune cell composition of the spleen and whole blood, as well as stem and progenitor cells of the bone marrow, were assessed by flow cytometry. Whole blood and bone marrow monocytes were stimulated with LPS and supernatant levels of TNF and IL-6 detected by ELISA. Furthermore, naïve bone marrow monocytes were analyzed for metabolic (Seahorse technology) and transcriptomic (RNA sequencing) changes. Flow cytometric analysis revealed an increase of inflammatory monocytes and regulatory T cells in the spleen, whereby immune composition of whole blood kept unchanged. Granulocyte-monocyte progenitor cells are increased in sepsis survivors. Systemic cytokine response was unchanged after LPS challenge. In contrast, cytokine response of post-septic naïve bone marrow monocytes was increased. Metabolic analysis revealed enhanced glycolytic activity, whereas mitochondrial indices were not affected. In addition, RNA sequencing analysis of global gene expression in monocytes revealed a sustained signature of 367 differentially expressed genes. We here demonstrate that sepsis via functional reprogramming of naïve bone marrow monocytes induces a cellular state of trained immunity, which might be counteracted depending on the compartmental localization of the cell. These findings shed new light on the complex aftermath of sepsis and open up a new pathophysiological framework in need for further research.

## Introduction

The immune system is historically divided into an innate and an adaptive branch. Adaptive immune responses, involving lymphoid immune cells, are slow, but specific for certain pathogens. Cells of the adaptive immune system are able to build up an organism-level immune memory as a prerequisite to respond faster to a second encounter (1). In contrast, innate immune responses representing the first line of host defense against invading pathogens are mediated by cells of the myeloid lineage with a fast kinetic and pathogen-unspecific recognition of conserved patterns (2). Further, they were classically proposed to lack memory function (3). In the last decades, this concept was counteracted. It has been shown that an activation of innate immune cells is believed to leave “immunological scars” on the cellular level, leading to either an boosted (training) or a diminished (tolerance) response to a secondary stimulus. This phenomenon of cellular adaption was termed “innate immune memory” (4–6).

Mechanisms involved in the regulation of this long-term innate immune memory is regulated are still under consideration. Recent studies demonstrate the involvement of metabolic reprogramming in this process (7, 8). For example, as a central mechanistic prerequisite of trained immunity, the shift from oxidative phosphorylation toward aerobic glycolysis has been proposed (Warburg effect) (9, 10). Furthermore, not only glucose metabolism but also such pathways as glutaminolysis or cholesterol synthesis were shown to play critical roles in the induction of a trained state (11, 12). Underlying these metabolic changes, epigenetic reprogramming is considered to be the main regulator of trained immunity (13). Concerning the short half-life of innate immune cells, especially monocytes in the circulation, a reprogramming of hematopoietic progenitor cells in the bone marrow was shown to be involved in the long-term maintenance of those effects (13–15).

Sepsis is a life-threatening syndrome, triggered by an infection which initiates a strong systemic release of inflammatory mediators. This cytokine storm during the acute phase of sepsis can lead to hypotension, cardiovascular dysfunction, tissue damage, and multiple organ failure. Simultaneously, an output of anti-inflammatory cytokines occurs to restrict the damage of the inflammatory reaction (16, 17). In the late phase of sepsis, the latter reaction overwhelms, counteracts host's initial response and can lead to a systemic state of immune tolerance. This so-called sepsis-induced immunosuppression can persist for years, rendering patients susceptible to persistent and secondary nosocomial infections, associated with an increased mortality rate (16–19).

Since there is growing evidence that innate immune memory plays a crucial role in the maintenance of post-septic immunosuppression (20), we used a polymicrobial animal model of sepsis to shed light on this point. We here prove against our expectations that sepsis induces a trained immunity phenotype in naïve bone marrow monocytes months after the initial insult, whereas the systemic response remained unaltered.

## Materials and methods

### Mice

All animal procedures were conducted in accordance with the German Animal Welfare Act law and were approved by the regional council Karlsruhe (reference number G-132/15).

Twelve week old male C57BL/6 mice were purchased from Janvier Laboratories (Le Genest Saint Isle, France). All animals were housed in a 12 h light/dark cycle at 22°C, receiving food and water *ad libitum*. Mice were acclimatized for 7 days before conduct of any experimental procedure.

### Polymicrobial sepsis model

**Figure 1:**
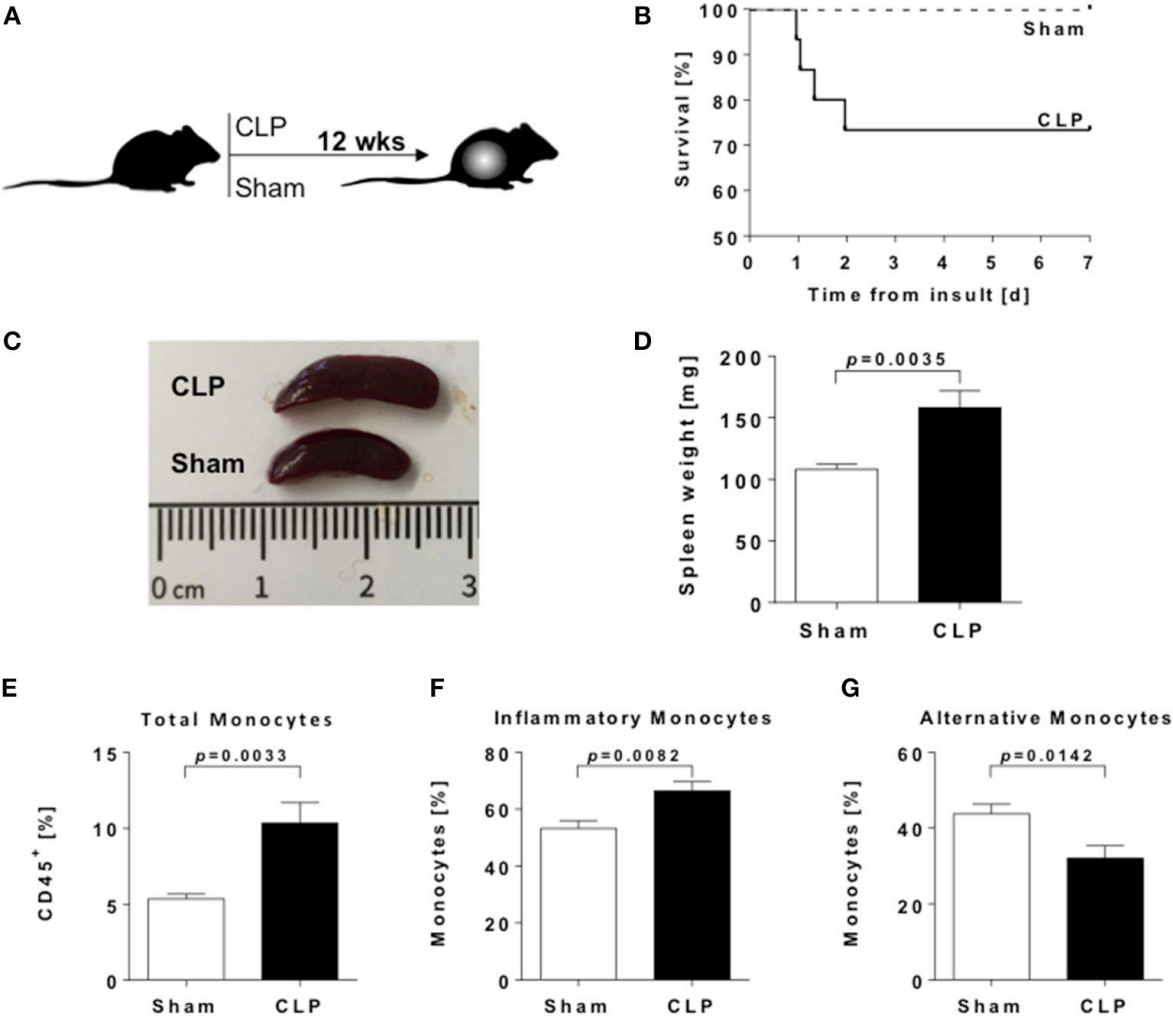
Splenomegaly and enhanced frequencies of splenic myeloid cells in sepsis-survivors. **(A)** Experimental design of the study. **(B)** Survival rate of male C57BL/6 mice after CLP (*n* = 15; solid line) or sham (*n* = 9; dashed line) procedure. **(C)** Representative spleens and **(D)** spleen weights from CLP (black bar) and sham (open bar) animals twelve weeks postoperative (*n* = 9 per condition). Spleens were dissected and immune cell populations measured by flow cytometry. Frequencies of **(E)** total monocytes (CD11b^+^ F4/80^−^), **(F)** inflammatory (CD11b^+^ F4/80^−^ Ly6C^+^), and **(G)** alternative monocytes (CD11b^+^ F4/80^−^ Ly6C^−^). Data are represented as mean ± SEM, *n* = 8 per condition.

For the investigation of post-septic immunological consequences a cecal ligation and puncture (CLP) mouse model was used as described before (21). Briefly, mice were anesthetized by an intraperitoneal injection of 100 mg/kg ketamine (Ketanest®S, Pfizer Pharma, Berlin, Germany) and 20 mg/kg xylazine (Xylavet, CP-Pharma, Burgdorf, Germany). After median laparotomy, cecum was mobilized, ligated (5 mm).and punctured once with a 23 G needle (BD Microlance™ 3, BD Medical, Heidelberg, Germany). Fecal content was gently extruded and the cecum afterwards relocated. Mice were supplemented with 400 μL 0.9% sodium chloride (B. Braun, Melsungen, Germany), given directly in the abdominal cavity and abdomen was closed thereafter with a double suture. Control animals received a sham surgery without cecal ligation and puncture. For pain relieve, mice were treated with 0.05 mg/kg bodyweight buprenorphine (Temgesic, RB Pharmaceuticals, Slough, UK) every 8 h for 2 days after surgery. In total, 15 animals received a CLP and 9 animals a sham surgery. All animals were subsequently housed till euthanasia 12 weeks after intervention (Figure 1A).

### Cell isolation

Mice were euthanized via cardiac puncture. Whole blood was collected in heparinized (Heparin-Natrium-25000-Ratiopharm, Ulm, Germany) syringes (BD Plastipak, BD Medical, Heidelberg, Germany).

Spleen was dissected and weighed. For dissociation of splenic tissue, the spleen was passed through a 70 μm cell strainer (Greiner BioOne, Frickenhausen, Germany). Cells were centrifuged and resuspended in ACK lysis buffer [0.15 M NH_4_Cl (Riedel-de-Haen, Seelze, Germany), 1 mM KHCO_3_ (Merck, Darmstadt, Germany), 0.1 mM Na_2_EDTA (Life Technologies, Darmstadt, Germany)] for red blood cell lysis. After centrifugation, pellet was resupended in PBS and cells counted.

Bone marrow was extracted from femurae and tibiae of CLP and control mice as follows. Bones were prepared by removing all soft tissue without fracturing, followed by a decapping of the bone on both sides. Subsequently, bone marrow was flushed out with pre-warmed RPMI 1640 media (Life Technologies, Darmstadt, Germany) supplemented with 10% Ultra-low endotoxin FCS (Cell concepts, Umkirch, Germany). 10^7^ cells were set aside for flow cytometric analysis.

Naïve bone marrow monocytes were isolated by negative selection using magnetic bead-based MACS technology (Monocyte Isolation Kit (BM), Miltenyi Biotec, Bergisch Gladbach, Germany) according to the manufacturer's instructions. Briefly, bone marrow cells were incubated with FcR Blocking Reagent and Monocyte Biotin-Antibody Cocktail. After washing, cells were incubated with Anti-Biotin MicroBeads and separation was facilitated on an AutoMACS Pro separator (Miltenyi Biotec, Bergisch Gladbach, Germany) via depletion of labeled non-monocyte cells. Quality control was performed by flow cytometry.

### Flow cytometry

Whole blood (50 μl) was incubated with CD45 Pacific Blue (BioLegend, San Diego, USA), CD11b FITC (BioLegend, San Diego, USA), F4/80 PE (BioLegend, San Diego, USA), and Ly6C PerCP-Cy5.5 (BD Biosciences, Heidelberg, Germany) for identification of myeloid cells and with CD45 APC-Cy7 (BioLegend, San Diego, USA), CD4 PerCP-Cy5.5 (BioLegend, San Diego, USA), CD25 PE (BioLegend, San Diego, USA), and CD127 APC (BioLegend, San Diego, USA) for identification of regulatory T cells. Incubation was done for 30 min at 4°C in the dark. Subsequently, erythrocytes were lysed by adding FACS lysing solution (BD Biosciences, Heidelberg, Germany) and cells analyzed on a FACSverse flow cytometer (BD Biosciences, Heidelberg, Germany).

Splenic cells (10^6^ cells per tube) were stained with CD45 APC (BioLegend, San Diego, USA), CD4 PerCP/Cy5.5 (BD Biosciences, Heidelberg, Germany), CD25 PE (BioLegend, San Diego, USA), CD127 APC (BioLegend, San Diego, USA), CD3e FITC (BD Biosciences, Heidelberg, Germany), CD8a PE/Cy7 (BioLegend, San Diego, USA) and CD19 eFluor 450 (Thermo Fisher Scientific, Dreieich, Germany) for regulatory T cells, CD45 eFluor450 (Thermo Fisher Scientific, Dreieich, Germany), CD11b FITC (BD Biosciences, Heidelberg, Germany), F4/80 PE (BioLegend, San Diego, USA), Ly6C PerCP/Cy5.5 (BioLegend, San Diego, USA), and CD11c (BioLegend, San Diego, USA) for myeloid cells. FMO control for Ly6C was used for proper gate placement.

For the analysis of hematopoietic progenitor cell composition in bone marrow, isolated cells were stained with Lineage Pacific Blue (Biolegend, San Diego, USA), Sca-1 PE/Cy7 (Biolegend, San Diego, USA), CD117 (c-Kit) APC (Biolegend, San Diego, USA), CD34 FITC (Miltenyi Biotec, Bergisch Gladbach, Germany), CD16/32 PE (Miltenyi Biotec, Bergisch Gladbach, Germany), CD48 APC/Cy7(Biolegend, San Diego, USA), and CD150 PerCP/Cy5.5 (Biolegend, San Diego, USA). FMO control tubes for CD34, CD16/32 and CD150 were included for proper gate adjustment. Cells were fixed with 4% PFA and detection was performed on a FACSverse flow cytometer (BD Biosciences, Heidelberg, Germany).

Quality control was performed via flow cytometry. Therefore, bone marrow monocytes (1·10^5^ cells) were centrifuged and stained with CD11b PE antibody (BioLegend, San Diego, USA) and Ly6C FITC antibody (BioLegend, San Diego, USA).

### *Ex vivo* stimulation and cytokine analysis

Heparinized whole blood from left-ventricular cardiac puncture was transferred into a 96-well plate (Sarstedt, Nümbrecht, Germany) and diluted 1:1 with RPMI 1640 Media (Life Technolgies, Darmstadt, Germany) supplemented with 10% Ultra-low endotoxin FCS (Cell Concepts, Umkirch, Germany). Stimulation was performed with 200 ng/mL LPS (O111:B4, Ultrapure, Invivogen, Toulouse, France) or solvent as control.

Isolated monocytes were counted using a Scepter™ 2.0 cell counter (Merck, Darmstadt, Germany). 2 × 10^5^ cells were seeded into a 96-well plate (Sarstedt, Nuembrecht, Germany) with RPMI 1640 Media (Life Technologies, Darmstadt, Germany) supplemented with 10% Ultra-low endotoxin FCS (Cell Concepts, Umkirch, Germany). After 1 h rest, cells were stimulated as described above.

After incubation for 24 h (37°C, 5% CO_2_), the supernatants from whole blood as well as monocytes were collected and TNF-α and IL-6 concentrations were determined using Mouse TNF-α and IL-6 DuoSet ELISA (R&D Systems, Minneapolis, USA), respectively, according to manufacturer's instruction.

### Extracellular flux analysis

Mitochondrial function (oxidative phosphorylation) and glycolytic rate of naïve bone marrow monocytes were assessed using a modified variant of the XFp Cell Mito Stress Kit and a Seahorse XFp Analyzer (both Agilent Technologies, Waldbronn, Germany). In brief, monocytes were washed with Seahorse XF Base Medium (Agilent Technologies, Waldbronn, Germany) supplemented with 10 mM glucose (Sigma Aldrich, Taufkirchen, Germany), 5 mM HEPES (Agilent Technologies, Waldbronn, Germany), 2 mM glutamine (Life Technologies, Darmstadt, Germany) and 1 mM pyruvate (Life Technologies, Darmstadt, Germany), and seeded in Cell-Tak (Corning, Kaiserslautern, Germany) coated 8-well Seahorse plates (1.5 × 10^5^ cells per well). All experiments were performed with two technical replicates. Oxygen consumption rate (OCR) and extracellular acidification rate (ECAR) were measured consecutively three times under basal conditions and after sequential injection of Oligomycin A (1 μM), FCCP (2 μM), Rotenone/Antimycin A (0.5 μM; all three reagents included in XFp Cell Mito Stress Kit, Agilent Technologies, Waldbronn, Germany) and finally 2-Deoxyglucose (50 mM) (Sigma Aldrich, Taufkirchen, Germany). Evaluation and calculation of mitochondrial and glycolytic indices was done using Wave software (Agilent Technologies, Waldbronn, Germany).

### RNA-Seq

RNA from naïve bone marrow monocytes was isolated using RNeasy Micro Kit (Qiagen, Hilden, Germany) according to the manufacturer's instructions after lysis with RLT Buffer containing 1% β-mercaptoethanol (Roth, Karlsruhe, Germany) and homogenization using QIAshredder (Qiagen, Hilden, Germany). Concentration and purity was determined with a Nanodrop 2000 spectrophotometer (Thermo Fisher Scientific, Dreieich, Germany) and integrity of RNA was assessed using the RNA 6000 Nano Kit and a Bioanalyzer (both Agilent Technologies, Waldbronn, Germany). RNA-seq including sample preparation, library preparation, and Illumina sequencing was performed by GATC Biotech AG (Konstanz, Germany). Raw sequencing data of individual samples is publicly available from the NCBI BioProject repository (https://www.ncbi.nlm.nih.gov/bioproject/) under the BioProject ID PRJNA488339.

### RNA-Seq reads processing, mapping

Initial quality control using FastQC (22) was performed for the available RNA-seq datasets. Subsequent processing included filtering with SortMeRNA (23) to remove contaminants of ribosomal RNA as well as trimming of short or low quality reads and TruSeq adapter sequences by Trimmomatic (24) software.

For main processing, the remaining reads were mapped to *Mus musculus* release M17 (GRCm38.p6) reference genome available from the GENCODE project (https://www.gencodegenes.org) using STAR (24) alignment software. Comprehensive gene annotation on the primary assembly (chromosomes and scaffolds) was chosen as superset of the main annotation. Unambiguously mapped and unique reads were kept. SAMtools (25) was used to convert the resulting sequence alignment maps to sorted binary alignment format (BAM) for downstream analysis.

### Differential expression, gene-ontology term analyses

Feature counting was performed using HTSeq (26) for all replicates against the respective GRCm38.p6 gene transfer file. Since all samples were represented by two technical replicates, count data was initially merged per sample to conserve existing counts while maintaining independence of the available biological replicates. DESeq2 (27) was used for differential expression analysis of count data. The differentially expressed genes as identified by DESeq2 were filtered to results with absolute linear fold change values above 1.5 and *p*-values below 0.02.

Over-represented GO-terms were identified by use of Genomatix Genome Analyzer (Genomatix, Munich, Germany) separately for both up- and down-regulated gene sets.

### Heatmap generation, principle component analysis

For heatmap visualization, library-size normalized count data of filtered differentially expressed genes were selected. To generate an informative heatmap, normalized count values per gene were standardized to z-scores. The final heatmap was clustered per gene as well as per experimental sample based on Ward's hierarchical agglomerative clustering method (Euclidean distance measure; Ward2 criterion).

Principle component analysis was performed on the standardized library-size normalized count data for differentially expressed genes.

### Statistics

Statistical data analysis was carried out in GraphPad Prism (V6.0 for Windows, GraphPad Software, La Jolla, USA). For group comparisons, unpaired *t*-test (two-tailed) was performed. Bar-graph data is represented as mean ± SEM. Survival rate is represented as Kaplan-Meier curve. For all analysis, statistical significance was assumed at *p* < 0.05.

## Results

### Sepsis induces sustained alterations of spleen morphology and cell composition

To investigate the long-term consequences of sepsis on innate immunity, we used the polymicrobial CLP mouse model. Overall mortality rate of the CLP group was 28% (4/15 animals), while no animal of the control group (Sham) died (Figure 1B). Enhanced loss of body weight and a higher clinical score in the CLP group indicates the successful induction of sepsis (Supplementary Figures 1A,B).

Twelve weeks after CLP or Sham surgery, we found markedly enlarged spleens with higher weights in post-septic animals (Figures 1C,D). Frequencies of monocyte populations were analyzed by flow cytometry (Supplementary Figure 1C). We found a significant increase of frequencies in total CD11b^+^ F4/80^−^ splenic monocytes after sepsis (Figure 1E). We further distinguished monocytes due to their functional properties: Ly6C^+^ inflammatory monocytes (Figure 1F) were significantly increased, whereas Ly6C^−^ alternative monocytes (Figure 1G) were significantly decreased in the post-septic spleen. Frequencies of splenic CD11b^+^ F4/80^+^ macrophages were enhanced as well (Supplementary Figure 1D).

Further evaluation of CD4^+^ CD25^+^ CD127^dim^ regulatory T cell (T_reg_) frequencies (Supplementary Figure 1E) revealed an increase of these immunosuppressive cells (Supplementary Figure 1F) as well.

### The systemic immune response is not altered in post-septic mice

**Figure 2:**
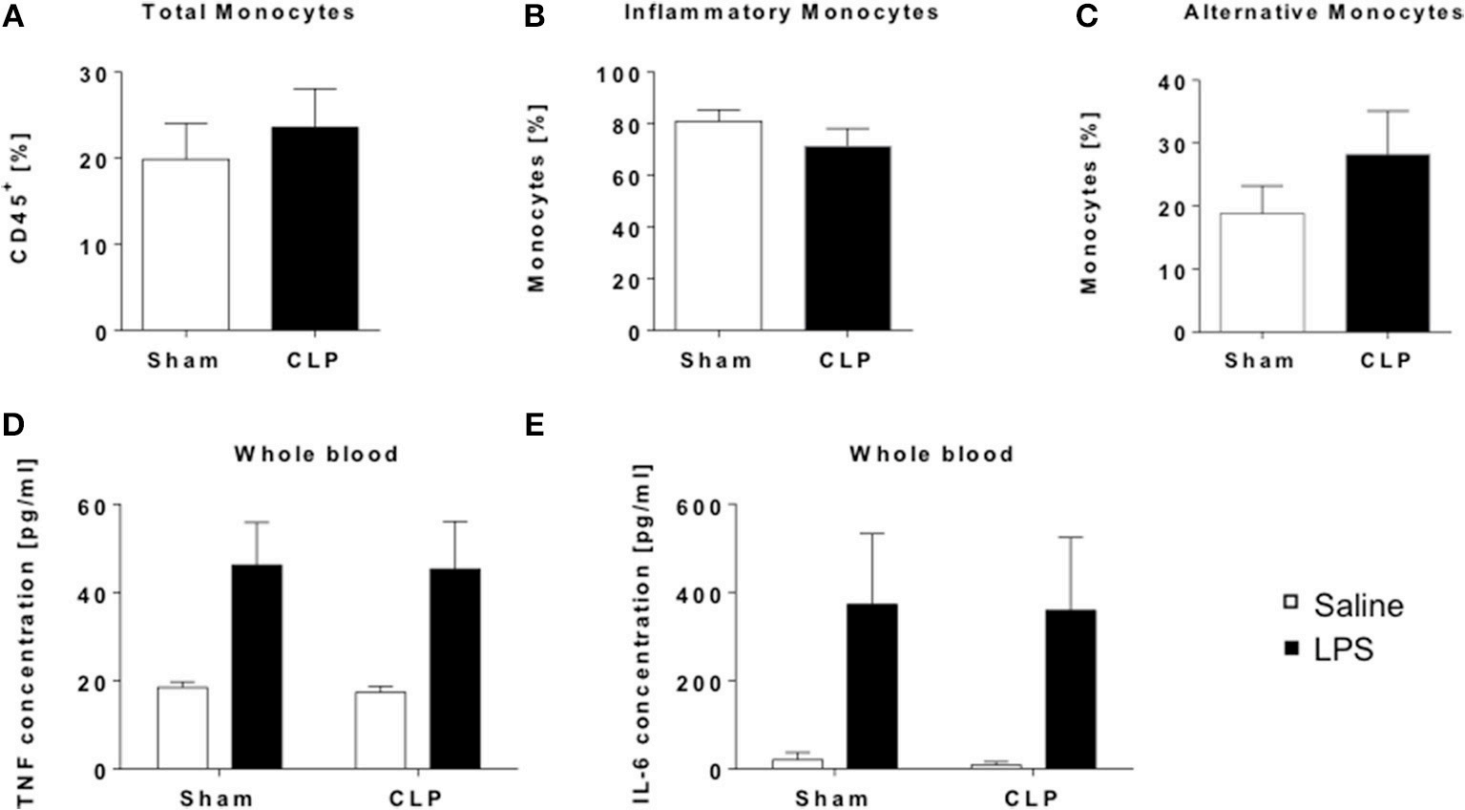
Whole blood analysis revealed no systemic alterations in the immune appearance of CLP mice. Immune cell composition of whole blood from CLP (black bar) and sham mice (open bar) was determined by flow cytometry. Frequencies of **(A)** total monocytes (CD11b^+^ F4/80^−^), **(B)** inflammatory (CD11b^+^ F4/80^−^ Ly6C^+^), and **(C)** alternative monocytes (CD11b^+^ F4/80^−^ Ly6C). For functionality assessment whole blood was stimulated with either LPS (black bar) or saline (open bar) for 24 h and cytokine levels in supernatants detected by ELISA: **(D)** TNF, and **(E)** IL-6. Data are represented as mean ± SEM, *n* = 9 per condition for FACS and *n* = 5 per condition for stimulation experiments.

Next we asked, if such a condition can be found in the blood as well. We performed flow cytometric analysis of monocyte as well as T_reg_ frequencies in whole blood (Supplementary Figures 2A,B). We found no significant change in either total monocyte frequency or the subgroups of inflammatory or alternative monocytes in survivors of sepsis (Figures 2A–C). Nevertheless, contrasting to the results in spleen, Ly6C^+^ inflammatory monocytes (Figure 2B) rather showed a decreasing trend, whereas Ly6C^−^ alternative monocytes (Figure 2C) were slightly increased in whole blood. T_Regs_ were also marginally increased after CLP (Supplementary Figure 2C), in line with the results of the spleen. We evaluated the overall responsivity of the blood cells upon LPS stimulation and found no differences in the TNF-α and IL-6 output between CLP and sham mice (Figures 2D,E).

Together, these results show that there is no long-term impact of sepsis on the systemic immune response in mice 12 weeks after insult.

### Sepsis induces a sustained shift toward an increased myeloid hematopoiesis

Bone marrow is largely constituted of hematopoietic stem cells and thereby the source of newly generated immune cells for the steady replenishment of circulating cells as well for as the recruitment to tissues during e.g., infection. Evidence is mounting that inflammatory processes impact monocyte development and alter cellular functions already on this level (28). We characterized hematopoietic and progenitor cells from whole bone marrow of post-sepsis and sham mice by flow cytometry (Supplementary Figure 3).

**Figure 3:**
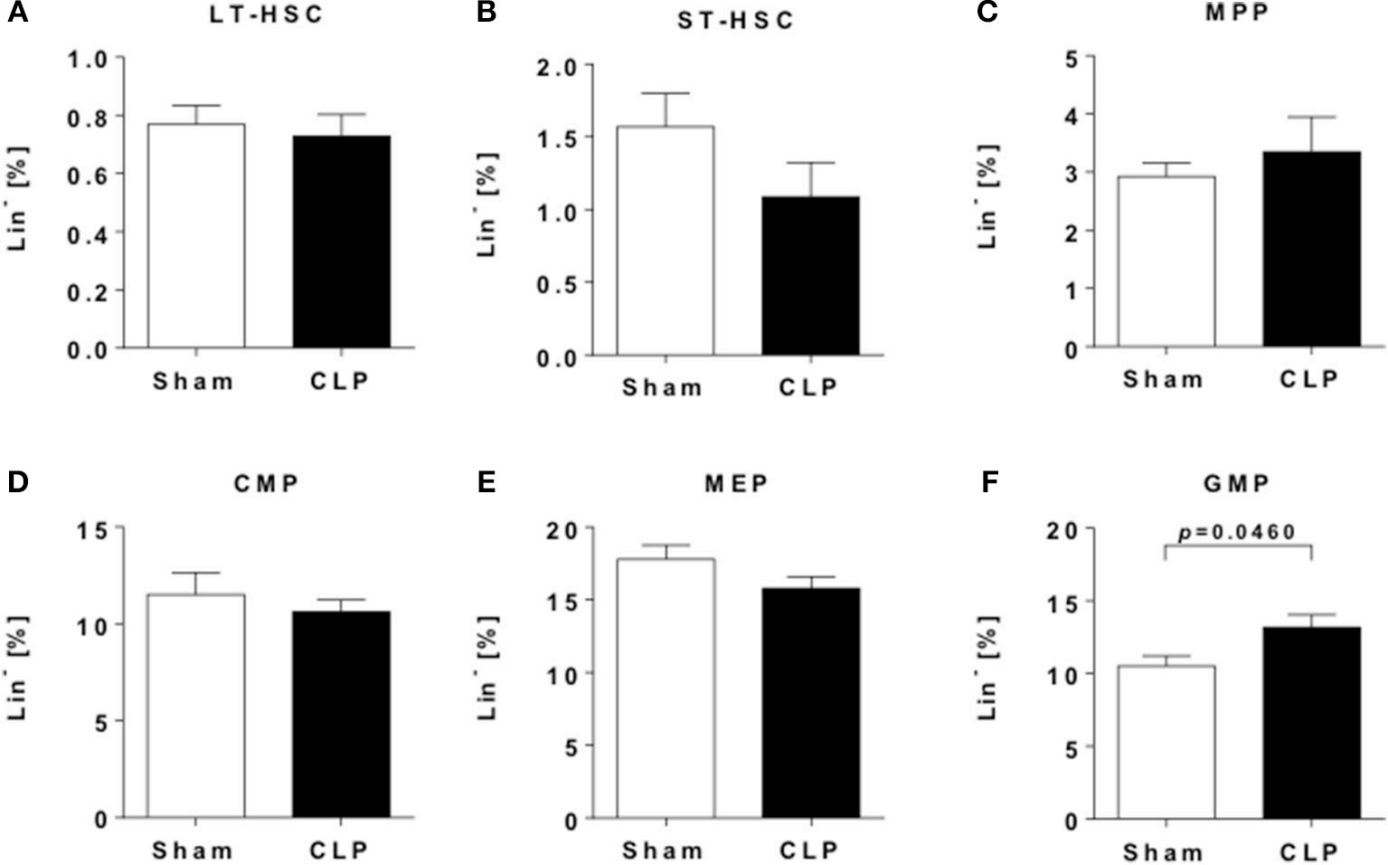
Sepsis induces an increase in myeloid progenitor cells. Hematopoietic progenitor and stem cells (HPSCs) from the bone marrow of CLP (black bar) and sham (open bar) mice were evaluated by flow cytometry. Frequencies of **(A)** LT-HSC (LSK CD48^−^ CD150^+^), **(B)** ST-HSC (LSK CD48^−^ CD150^−^), **(C)** MPP (LSK CD48^+^ CD150^−^), **(D)** CMP (LS^−^K CD16/32^int^ CD34^+^), **(E)** MEP (LS^−^K CD16/32^−^ CD34^−^), and **(F)** GMP (LS^−^K CD16/32^+^ CD34^+^). Data are represented as mean ± SEM, *n* = 9 per condition.

Twelve weeks after insult, no differences in frequencies of long-term hematopoietic stem cells (LT-HSCs) (Figure 3A) were observed, while frequencies of short-term hematopoietic stem cells (ST-HSCs) were slightly decreased (Figure 3B). Other progenitor cells populations, including multipotent and common myeloid progenitors (MPP/CMP) as well as megakaryocyte-erythrocyte progenitors (MEPs) (Figure 3E).did only show subtle differences between the groups (Figures 3C–E). In contrast, late-stage granulocyte-monocyte progenitors (GMP) are significantly enriched (Figure 3F), indicating a sustained shift to myelopoiesis.

### Naïve bone marrow monocytes of post-septic animals exhibit a trained immunity phenotype

**Figure 4:**
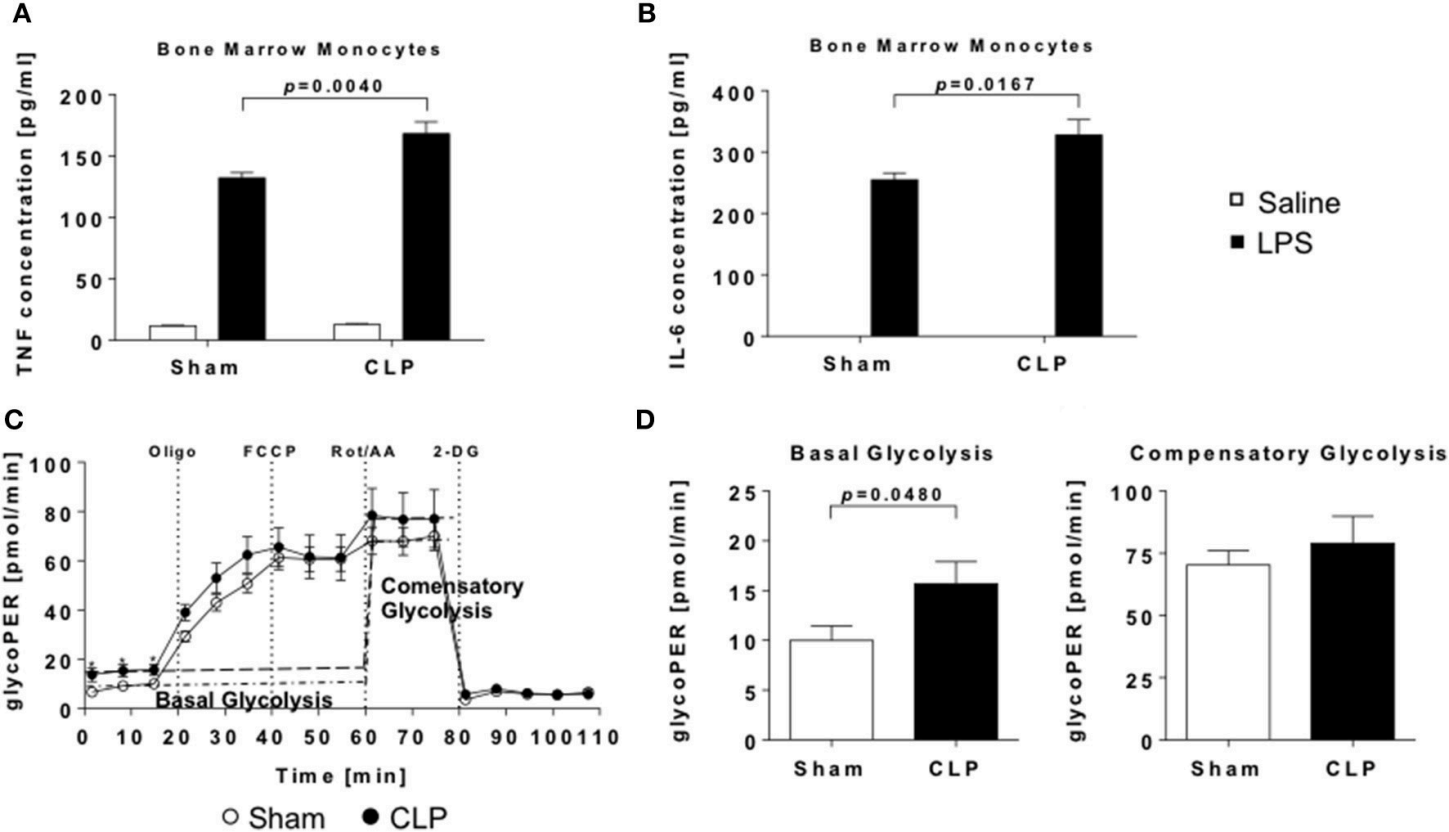
Enhanced inflammatory response to LPS and increased glycolytic activity of post-septic naïve bone-marrow monocytes. Monocytes were stimulated with LPS or saline for 24 h and levels of **(A)** TNF and **(B)** IL-6. in supernatants detected by ELISA. Metabolic activity of unstimulated monocytes was measured using Seahorse technology. **(C)** Cumulative glycolytic proton efflux rate and **(D)** evaluation of basal and compensatory glycolysis of bone marrow monocytes from CLP (black points/bars) or sham (open points/bars) mice. Data are represented as mean ± SEM, *n* = 9 per condition.

To evaluate if sepsis alters the function of naïve monocytes in a long-term manner, we stimulated MACS-sorted bone marrow monocytes with LPS and measured the production of TNF and IL-6. Compared to animals without experienced sepsis, supernatant concentrations of both cytokines were significantly elevated after stimulation (Figures 4A,B), resembling a typical hallmark of trained immunity.

An additional characteristic of trained immune cells is the shift of cellular metabolism from oxidative phosphorylation (OXPHOS) to aerobic glycolysis (7, 28). To proof, whether bone marrow monocytes are “trained” by sepsis, we conducted measurements of mitochondrial and glycolytic function by Seahorse. Basal glycolysis was significantly enhanced, while compensatory glycolysis was not elevated in CLP animals (Figures 4C,D). Also, indices of mitochondrial function were not altered (Supplementary Figure 4).

In summary, our finding of enhanced cytokine response as well as increased glycolysis is indicative for a trained state of naïve bone marrow monocytes in the post-septic mice.

### RNA-Seq analysis reveals long-term transcriptomic alterations in naïve bone marrow monocytes

**Figure 5:**
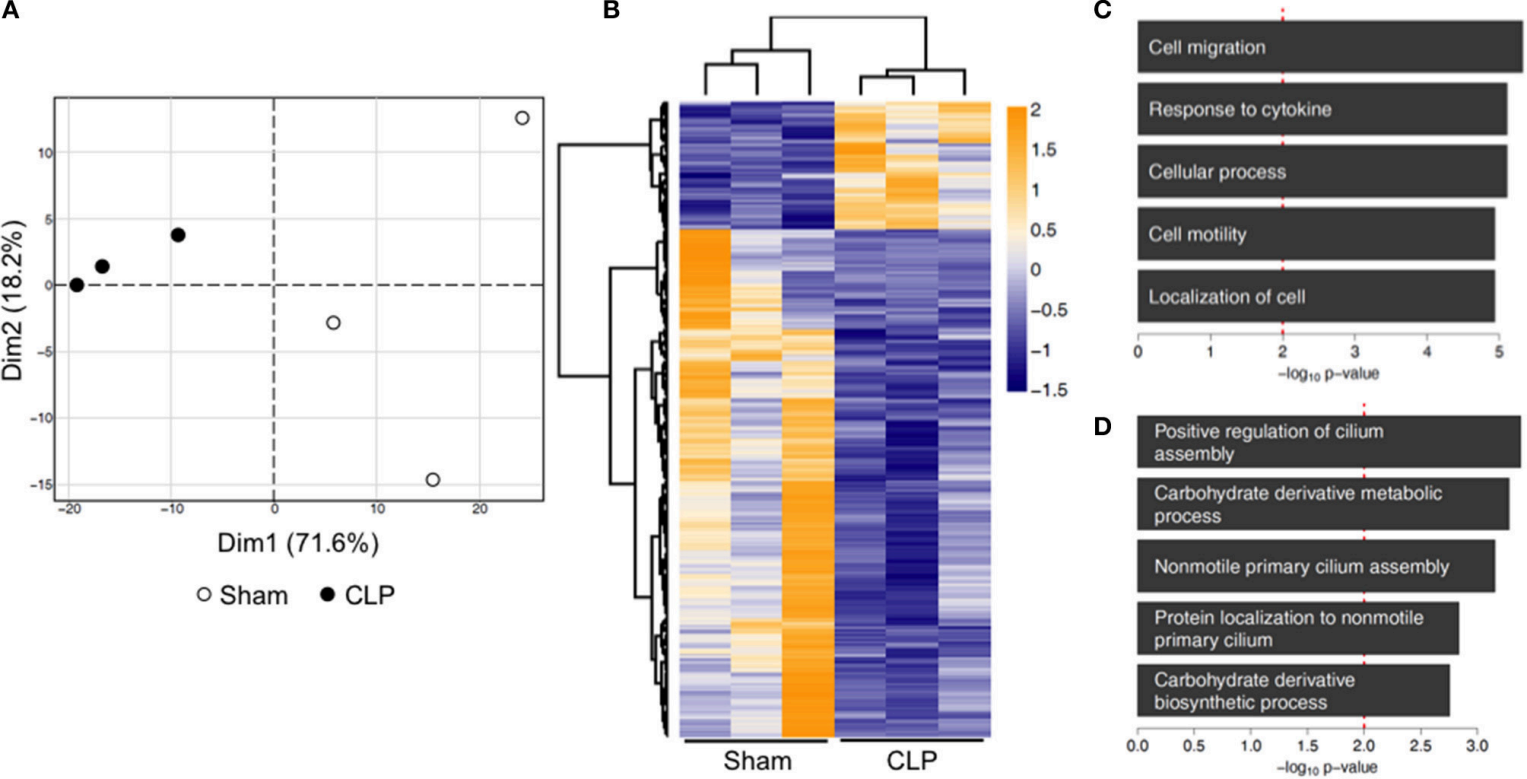
Transcriptional reprogramming of naïve bone-marrow monocytes 12 weeks post-CLP or sham. RNA of bone-marrow monocytes was isolated and whole genome transcriptome analysis performed by RNA-seq. **(A)** Principal component analysis of 367 genes identified as differentially expressed (linear change in expression of >1.5-fold; *p* < 0.02). **(B)** The heat map is representing standardized counts of differentially expressed genes in post-septic bone marrow monocytes. **(C)** Gene ontology term analysis, showing the top five upregulated and **(D)** downregulated biological processes. *n* = 3 per condition.

Transcriptional alterations were described to be involved in trained immunity (20). To elucidate a mechanistic background for our functional findings, we performed RNA-seq analysis of naïve bone marrow monocytes. We found a total of 367 differentially expressed genes in post-septic monocytes compared to control, from which 73 were up- and 294 down-regulated (linear expression changes >1.5-fold; *p* < 0.02) (full list available as Supplementary File 1). Principal component analysis of those 367 genes revealed a distinct separation of the animals according to their prior exposure (Figure 5A). Additionally, genes were analyzed for overrepresentation of biological functions. Upregulated genes were involved in processes like “cell migration” or “response to cytokines” (Figures 5B,C), whereas down-regulated genes were involved in “positive regulation of cilium assembly” or “carbohydrate derivative metabolic process” (Figures 5B,D).

Altogether our findings indicate an induction of trained immunity in naïve bone marrow monocytes as a long-term consequence of sepsis. Metabolic, as well as transcriptomic alterations, are involved in establishing this state.

## Discussion

In our study, we used an animal model of polymicrobial sepsis to investigate the consequences of a survived sepsis on innate immune cells, especially monocytes, in a clinically relevant timeframe.

The first finding was a persisting splenomegaly in animals after sepsis. Splenomegaly is typically associated with the expansion of immune cell populations and therefore an important feature of acute and chronic infections. We found increased frequencies of monocytes in post-septic spleens, with enhanced inflammatory and decreased alternative monocytes. Using a similar model, Valdes-Ferrer and colleagues found splenomegaly as well as increased frequencies of splenic monocyte populations within 10 days after CLP, but decreasing over (29). Contrasting to our findings, 12 weeks post-CLP both values returned back to baseline in their study. The opposing outcomes might be explained by the use of different mouse strains: Valdes-Ferrer used Th2-biased Balb/c mice, whereas we used Th1-biased C67BL/6 mice (30, 31).

Regulatory T cells are associated with immunosuppression and involved in the maintenance of self-tolerance (32). Our study shows enhanced frequencies of regulatory T cells in spleen and blood of animals 12 weeks after sepsis. Elevation of splenic regulatory T cells with higher repressive functionality in the post-septic environment has been described before in mice 15 days after CLP (33). This study further demonstrated that the expansion was caused by a dendritic cell mediated conversion of CD4^+^ CD25^−^ T cells to CD4^+^ CD25^+^ regulatory T cells. The co-occurrence of both inflammatory as well as immunosuppressive conditions hints toward a compartmentalized and cell-mediated counterbalance of innate immunity within the spleen.

Bone marrow is the central source of immune cells in the developed organism and is capable to respond to changing demands of the organism, e.g., during infection or injuries. Sepsis has been described to exhibit a suppressive influence on hematopoiesis, resulting in myelosuppression (34, 35). Opposing to these results, we found a significant increase of GMPs in post-septic animals, indicating a rather enhanced myelopoiesis. Moreover, we found slightly increased MPP populations, whereas ST-HSCs are marginally decreased. The controversial results compared to literature could be explained by different time points of assessment: both, the studies of Rodriguez et al. and Zhang et al. investigated HSPCs as early as 24 h after septic insult, a time point where early compensatory mechanism might still be present to counteract initial responses. Our investigation was performed long after insult, proving a sustained shift from myelosuppression to enhanced myelopoiesis, possibly as an adaptive mechanism after the clearance of the acute inflammatory tone of the system.

Our results are consistent with (36), who recently demonstrated enhanced myelopoiesis along with an increase of MPP populations as well as decreased ST-HSCs in animals after stimulation with β-glucan, the archetypical model of “trained immunity.” This effect diminished 28 days after stimulation, but GMPs remained significantly increased, similar to our results. These effects associated with trained immunity, induced by the stimulation with the *Candida albicans* cell wall component β-glucan can also be triggered by western diet, most probably due to a change in the microbiota composition in the gut (37). Considering our polymicrobal sepsis model, trained immunity could be induced by gastrointestinal-derived fungi, which enter circulation together with other bacterial pathogens via the extruded feces.

Since trained monocytes are characterized by an enhanced pro-inflammatory cytokine release after a second stimulus, we isolated bone marrow monocytes and analyzed their responsivity. We found an enhanced response after LPS stimulation, indicated by significantly increased level of TNF-α as well as IL-6 in the supernatant. Those functional results point toward a trained immune state after sepsis on the level of the bone marrow, similar to what has been described before for β-glucan stimulation (38, 39).

Another hallmark of trained immune cells is enhanced glycolysis, often described in combination with a reduction of oxidative phosphorylation and termed “aerobic glycolysis” (40, 41). We found indeed an increased glycolysis in monocytes of CLP animals, but could not determine any changes in oxygen consumption. A possible explanation for that phenomenon is, that monocytes investigated in our study were unstimulated, whereas innate immune cells described in metabolic studies of trained immunity were regularly analyzed after LPS re-stimulation. This second stimulus might be necessary for the full shift to aerobic glycolysis. Nevertheless, unstimulated cells seem to be already primed toward an enhanced glycolysis.

As described by several publications, transcriptional upregulation of genes involved in glycolysis as well as genes involved in mTOR (mammalian target of rapamycin) signaling, a key regulator of glucose metabolism, are responsible for the enhanced glycolytic activity (7, 11, 12, 36). To investigate, if post-septic bone-marrow monocytes show similar alterations, we performed whole-genome sequencing by RNA-seq. We found several genes linked to metabolic processes to be differentially expressed. For example, the gene encoding Ribulosephosphat-3-epimerase (RPE) was downregulated in monocytes of post-septic animals. RPE is involved in the regulation of the non-oxidative branch of the pentose phosphate pathway (PPP) and catalyzes the back reaction from PPP to glycolysis. Arts et al. (11) showed by NMR experiments with ^13^C-labeled glucose that the non-oxidative branch of PPP is inactive in trained immune cells. That seems to be induced by reduced RPE, driving PPP output toward purine metabolism.

Increased purine metabolism was described in trained immune cells to fulfill the increased nucleotide demand evoked by enriched transcription (8, 11). As described above, PPP output is skewed toward purine synthesis due to RPE downregulation. However, the gene *Nme7*, whose product catalyzes the transfer of phosphate from nucleoside triphosphates to nucleoside diphosphates and vice versa, is downregulated in post-septic monocytes. Also, we found genes involved in purine catabolism to be differentially expressed. For example, the enzymes Guanine deaminase (GDA) and Xanthine dehydrogenase (XDH) were upregulated. Both enzymes are involved in the degradation from guanine or hypoxanthine to xanthine and further to urate. Since we analyzed unstimulated cells, there is no acute need for enhanced purine synthesis, and therefore the overproduction resulting from the restriction of the non-oxidative PPP branch seems to be compensated by these gene expression changes. Further work is necessary to examine if the cells switch from purine catabolism to anabolism upon further immunological stimulation.

Another important metabolic pathway for the induction of trained immunity is glutaminolysis. Thereby, glutamine is converted via glutamate to α-ketoglutarate, an anaplerotic substrate of the tricarboxylic acid cycle (TCA). Enhanced glutaminolysis in trained immune cells replenishes the TCA cycle and increases amounts of the TCA metabolites succinate, fumarate, or malate. Fumarate itself was described to induce trained immunity by modulating epigenetic changes at gene promotors and therefore modulates the transcription of proinflammatory cytokines (11).

We did not find transcriptional changes directly correlated with glutaminolysis. Nevertheless, Histidine deaminase (HAL), an enzyme involved in the degradation from histidine to glutamate via urocanic acid, is upregulated in post-septic monocytes (42), representing a source of glutamate after a second pathogen challenge.

Immune regulatory genes involved in cytokine signaling are upregulated in monocytes of post-septic mice. One of those genes is myeloid differentiation protein 88 (MyD88). MyD88 is a crucial adapter protein involved in downstream signaling of several Toll-like receptors. Activation of MyD88 leads to the activation of NF-kappa-B and therefore to the induction of pro-inflammatory gene-transcription (43, 44). Another up-regulated adaptor, Janus kinase 3 (Jak3) is part of the Janus kinase/signal transducer and activator of transcription (JAK/STAT) pathway and regulates cytokine-mediated cellular responses, e.g., down-stream of the IL-6 receptor. JAK/STAT signaling is also involved in various developmental and homeostatic processes, including hematopoiesis and immune cell development (45–47). Upregulation of those genes can foster pro-inflammatory cytokine outflow after stimulation, leading to the increased TNF and IL-6 output as observed in our experiments.

Epigenetic reprogramming is also involved in the establishment and maintenance of trained immunity, but was not analyzed in our study (7, 13, 48). Therefore, this point is open for further investigations. Compared to other groups and earlier studies, we solely analyzed the molecular changes occurring in unstimulated monocytes. An evaluation of metabolic, transcriptional, and epigenetic alterations in post-septic naïve bone marrow monocytes after a second stimulus like LPS remains to be elucidated, especially considering a selective “training” for certain pathogens or stimuli. Also, the impact of the disease severity evoked during the model needs to be clarified, as our study made use of a “mild” CLP approach with only 25% lethality. Higher severity can be associated with a higher bacterial burden and might implicate divergent long-term consequences on immunity.

Several animal studies approached the concept of “post-septic immunosuppression” using double-hit models and combined an initial CLP (or pneumonia) with a second encounter by bacterial (e.g., *P. aeriugunosa* or *S. pneumoniae*) or viral (respiratory syncytial virus) pathogens. Most of those studies analyzed the susceptibility to a second stimulus only a few hours or days after the insult (49–52). Only Nascimento and colleagues demonstrated an enhanced vulnerability to opportunistic infections up to 30 days (50). Importantly, several of those studies are focused on the lung, thereby inducing secondary pneumonia and demonstrating the organ-specific induction of immune tolerance via e.g., TGF-β and accumulation of regulatory T cells (49–52). Especially, Roquilly et al. demonstrated in their pneumonia/pneumonia double-hit model the induction of local immune tolerance in bone marrow derived dendritic cells and macrophages after lung migration (52).

Our findings of a trained phenotype might seem contradictory on first sight, but actually, they complement the current state of knowledge regarding the long-term temporal dimension and indicate the inherent complexity of systemic diseases. Our study is the first approaching immune function 3 months after sepsis, resembling a clinically relevant post-ICU timeframe. One might hypothesize that after an early state of tolerance, as extensively proven before, the body overcompensates such state by acquiring a trained phenotype with increased myelopoiesis and cellular alterations, resembling potentially favorable long-term adaption of innate immunity. Considering the complex host response during sepsis with extensive crosstalk of organs, the possibility of a combined spatiotemporal impact, depending on both the site of initial infection as well as the time of observation is likely. Moreover, each model itself has several dimensions to be taken into account: from the age and strain of the mice, the characteristics, and quantity of the infectious agent, up to the evoked disease severity. In the future, mixed second hit models (abdomen/lung, lung/soft tissue, etc.) will be of tremendous importance to assess which (distant) organs and body compartments might have adapted and if this is detrimental or protective for the host. As mentioned before, organs like the lung itself adapt and generate an immunosuppressive milieu, which further shapes the invading cells function. Comparably, we did not observe alterations of the blood regarding an evoked cytokine secretion. The reasons for this surprising finding are elusive. However, upon release from the bone marrow, monocytes enter their short life cycle in blood, changing from inflammatory monocytes to alternative monocytes within a day before subsequently leaving circulation. We observed a slight increase in the frequency of alternative monocytes in the blood, hinting for a faster conversion rate or extended halftime of alternative monocytes until extravasation. Vice versa, inflammatory monocytes decrease, and this might blur the “trained” response observed in isolated monocytes of the bone marrow. Alternatively, persisting humoral factors like, e.g., metabolites might contribute to this peripheral phenomenon. Importantly, the question of adaption does not only extend solely to immune function, but rather to the fundamental functions of each organ or tissue.

In conclusion, the present study proves for the first time to our knowledge that sepsis induces a state of trained immunity both on a cellular level in naïve monocytes as well as in the hematopoietic niche of the bone marrow. These results refine the current hypothesis of a persisting global post-septic immunosuppression on the cellular level and therefore underline the requirement for further investigation of the immune system in sepsis survivors to understand the complex adaptions present.

## Author contributions

KB and FU planned and performed the animal experiments. KB, JS, IS, and DS conducted the analysis. KB, DS, MW, and FU performed data interpretation and statistics. KB, FU, and MW wrote the manuscript. All authors critically revised and drafted the submitted manuscript.

### Conflict of interest statement

The authors declare that the research was conducted in the absence of any commercial or financial relationships that could be construed as a potential conflict of interest.
