# Supplementary material for: Sepsis Induces a Long-Lasting State of Trained Immunity in Bone Marrow Monocytes
Source: Front Immunol. 2018 Nov 19;9:2685. doi: 10.3389/fimmu.2018.02685 (PMC6254543; doi:10.3389/fimmu.2018.02685)
Supplement: Supplementary file 1 [file Data_Sheet_1.pdf]

Supplem. Fig. 1

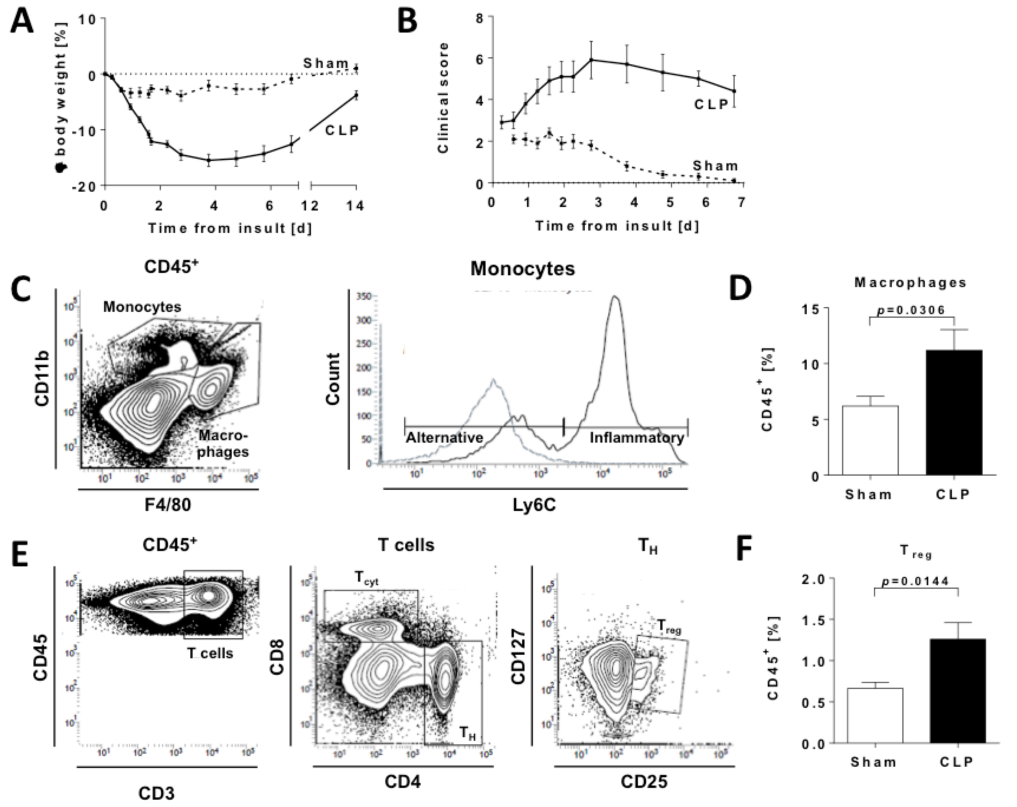

Supplementary Figure 1 (A) Weight loss and **(B)** clinical score of male C57BL/6 mice after CLP or sham surgery. **(C)** Representative flow-cytometric plots for the identification of splenic monocytes/macrophages. Spleen cells were gated for $CD45^+$ cells, followed by the differentiation between monocytes and macrophages via CD11b and F4/80. Monocytes were further characterized by Ly6C. FMO control for Ly6C is depicted as a gray dashed line. **(D)** Frequencies of splenic macrophages. **(E)** Representative plots for determination of splenic regulatory T cells. $CD45^+$ spleen cells were gated for T cells ($CD3^+$), followed by gating cytotoxic T cells (T_{cyt}; $CD3^+$ $CD8^+$) and T helper cells (T_H; $CD3^+$ $CD4^+$). Regulatory T cells (T_{reg}; $CD4^+$ $CD127^-$ $CD25^+$) were gated from T helper cells. F. Frequencies of splenic T_{reg}. Data are represented as mean ± SEM, n = 8 per condition.

Supplem. Fig. 2

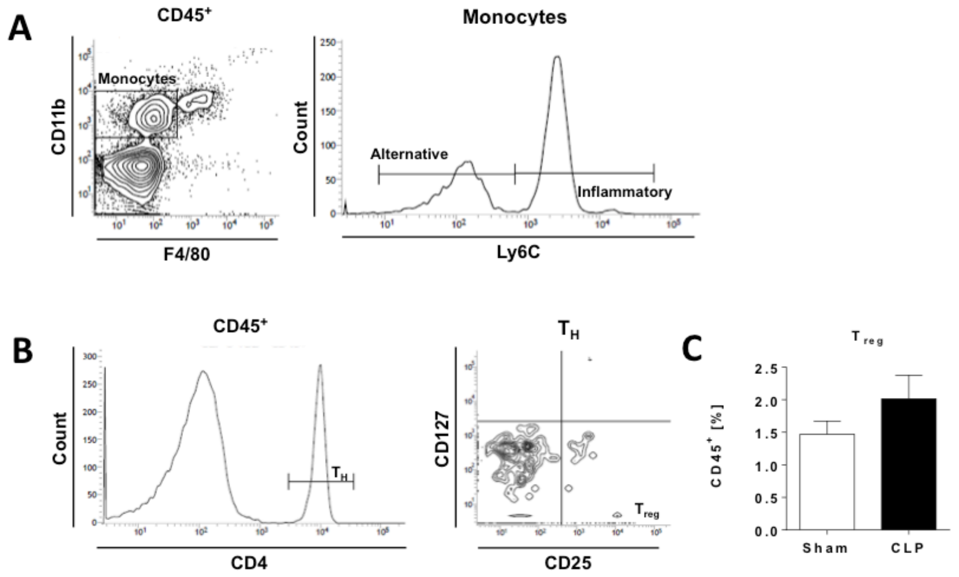

Supplementary Figure 2 (A) Representative gating strategy for identifying blood monocytes and their subpopulations. Whole blood cells were gated for $CD45^+$. Monocytes were identified via CD11b and F4/80 and further characterized by Ly6C. **(B)** Representative plots for identifying whole blood regulatory T cells. Whole blood cells were gated for $CD45^+$, followed by identification of T helper cells (T_H; $CD4^+$). Regulatory T cells (T_{reg}; $CD4^+$ $CD127^-$ $CD25^+$) were gated from T helper cells. **(C)** Frequencies of whole blood T_{reg}, in post-CLP (black bars) or sham (open bars) mice. Data are represented as mean ± SEM, n = 9 per condition.

Supplem. Fig. 3

A

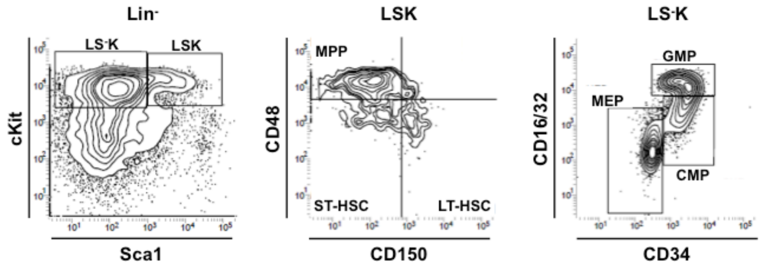

Supplementary Figure 3 Representative gating strategy for flow cytometry bone marrow analysis. Whole bone marrow cells were gated for lineage negative cells, followed by the discrimination of LSK ($Lin^- Sca1^+ cKit^+$) and LS^-K ($Lin^- Sca1^- cKit^+$) cells. LSK cells were further distinguished in multipotent progenitor cells (MPP), short-term HSC (ST-HSC) and long-term HSC (LT-HSC) by CD48 and CD150. LS-K cells are further characterized as common myeloid progenitors (CMP), megakaryocyte-erythrocyte progenitors (MEP) and granulocyte-macrophage progenitors (GMP) by CD16/32 and CD34.

Supplem. Fig. 4

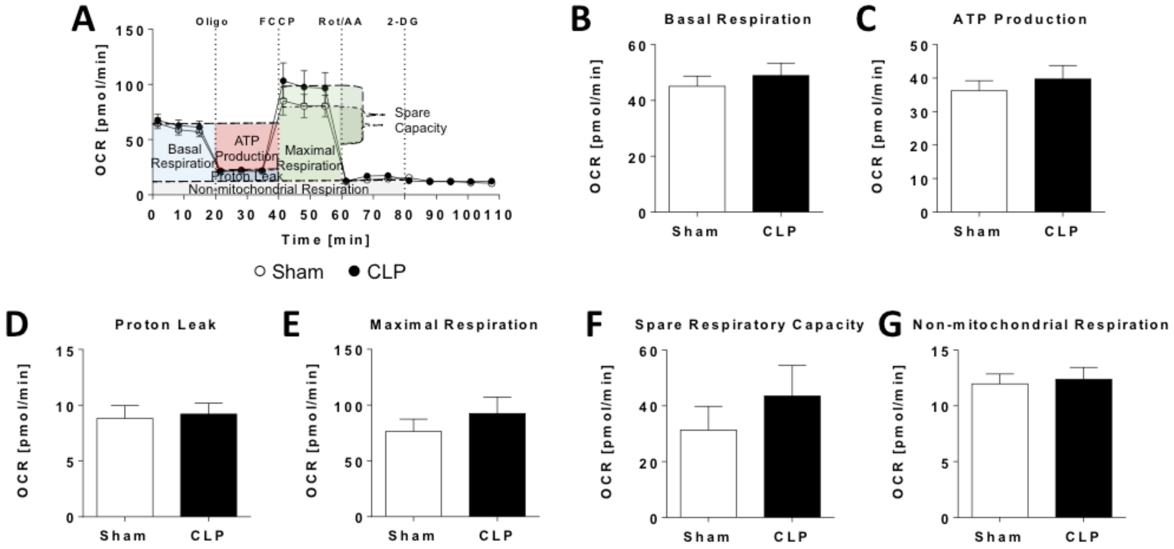

Supplementary Figure 4 (A) Representative Seahorse measurement of oxygen consumption rate (OCR), extracellular acidification rate (ECAR) and proton efflux rate (PER) of naïve bone marrow monocytes from post-CLP (black points) and sham (open points) mice. **(B)** Basal respiration, **(C)** ATP production, **(D)** proton leak, **(E)** maximal respiration, **(F)** spare respiratory capacity and **(G)** non-mitochondrial respiration of CLP (black bar) or sham mice (open bars). Data are represented as mean ± SEM, n = 9 per condition.
